# Aetiology of anaphylaxis in patients referred to an immunology clinic in Colombo, Sri Lanka

**DOI:** 10.1186/s13223-018-0295-0

**Published:** 2018-11-08

**Authors:** Nilhan Rajiva de Silva, W. M. D. K. Dasanayake, Chandima Karunatilake, Geethani Devika Wickramasingha, B. D. De Silva, Gathsauri Neelika Malavige

**Affiliations:** 1 Department of Immunology, Medical Research Institute, Colombo 08, Sri Lanka; 2 Institute of Biochemistry Molecular Biology & Biotechnology, University of Colombo, Colombo, Sri Lanka; 3 Department of Microbiology, Faculty of Medical Sciences, University of Sri Jayewardenepura, Colombo, Sri Lanka

**Keywords:** Anaphylaxis, Food allergy, Insect allergy, Drug allergy

## Abstract

**Background:**

The aetiology of anaphylaxis differs according to types of foods consumed, fauna and foliage and cultural practices. Although the aetiology of anaphylaxis in Western countries are well known, the causes in South Asian countries have not been reported. We sought to determine the causes of anaphylaxis in patients referred to an immunology clinic in Colombo, Sri Lanka.

**Methods:**

238 episodes of anaphylaxis were reviewed in 188 patients who were referred and skin prick tests and in vitro tests (ImmunoCap) were carried out to assess the presence of allergen specific IgE. Clinical features and severity of anaphylaxis was also recorded along with treatment received.

**Results:**

Anaphylaxis to food either following direct exposure 90/238 (37.5%) or after exercise in the form of food dependent exercise induced anaphylaxis 29/238 (12.2%) was the predominant cause of anaphylaxis. Allergy to cow’s milk and red meat, after immediate exposure, accounted for 66/238 (27.7%) of instances of all episodes of anaphylaxis and 66/90 (73.33%) of anaphylaxis due to food. Vaccines accounted for 28/238 (11.8%) of instances of anaphylaxis, especially among children. Of those who developed anaphylaxis to the MMR (n = 14), 71.4% of them had specific IgE to cow’s milk and 35.7% of them had specific IgE to beef. Of those who developed anaphylaxis to insect stings, 27/42 of these episodes occurred following stings of ants (family Formicidae). The predominant cause of anaphylaxis changed with the age, with food allergy being the most frequent trigger of anaphylaxis in childhood, while drug allergy and idiopathic anaphylaxis being more frequent after 30 years of age.

**Conclusions:**

In this cohort, anaphylaxis to red meat appears to be the predominant cause of food induced anaphylaxis and presence of beef specific IgE and cow’s milk, appears to be a predisposing factor for vaccine induced anaphylaxis.

## Background

Anaphylaxis is a potentially fatal allergic reaction, which occurs due to generalized mast cell degranulation. The prevalence and severity of all allergic diseases including anaphylaxis is on the rise [1, 2]. The main causes of anaphylaxis are food, drugs and insect venom allergy. Food allergy is the cause of anaphylaxis in approximately half of those experiencing anaphylaxis [2, 3]. Tree nuts, peanuts, cow’s milk, sea food and wheat are the main food allergens worldwide, although the type of food allergens responsible for anaphylaxis varies from country to country, possibly due to the differences in the type of food consumed [3].

Non-steroidal anti-inflammatory drugs (NSAIDs) is the leading cause of drug induced anaphylaxis [4]. Drugs account for approximately 1/3 of cases of anaphylaxis, but are responsible for nearly 60% of cases of fatal anaphylaxis [5]. The most common cause of drug induced fatalities is antibiotics followed by radio contrast media [5]. Insect venom allergy is an important cause of anaphylaxis especially in adult patients. Although Hymenoptera venom allergy due to wasp and bee stings are the main cause of insect venom anaphylaxis worldwide and is also reported in Sri Lanka [6, 7], anaphylaxis due to several ant species appear to be more frequent in this country (personal communication).

Most of the epidemiological data on anaphylaxis are from Western countries [3, 5, 8], where weaning practices, types of food consumed, exposure to insects and host genetics are very different. Data on the aetiology of anaphylaxis from South Asian countries is not available. The demographics or aetiology of anaphylaxis in Sri Lanka has not been systematically studied with the only available data being the aetiology of anaphylaxis due to specific triggers such as food dependent exercise induced anaphylaxis (FDEIA) and anaphylaxis to vaccines [9, 10]. Since the prevalence of allergy related diseases are reported to be on the rise in many countries including Sri Lanka [11], the study of causes of anaphylaxis in South Asian countries such as Sri Lanka is necessary. This data would be helpful in formulating health policies and planning of health care services.

## Methods

Patients with a history of anaphylaxis who were referred to the Immunology Clinic at the Medical Research Institute Colombo, Sri Lanka, from 2012 to 2017 were included in the study following informed written consent. Ethics Clearance was obtained from the Ethics Review Committee, Medical Research Institute (No: 39—2015). A detailed interviewer administered questionnaire was used to record the clinical symptoms and possible triggers of anaphylaxis. Anaphylaxis was diagnosed based on the WAO 2011 diagnostic criteria [4], using the patient’s symptoms and clinical records obtained at the time of referral.

### Identification of possible food allergens

Skin prick testing with the commercial allergens or prick to prick testing were carried out according to the guidelines set by the European Academy of Allergy and Clinical Immunology [12] when food allergy was suspected. A positive and a negative control were included in all instances. The size of the wheal was recorded at 20 min and a test was considered positive if the wheal size was > 3 mm of the negative control. Food specific IgE was determined using the Phadia ImmunoCap system in patients where a skin prick test was not performed due to non-availability of specific reagents. A positive IgE titre was defined as specific IgE levels of > 0.35 kUA/L. The patients were considered to have had anaphylaxis to a particular food, if the episode of anaphylaxis was triggered by immediate exposure to the food, and also if the presence of food specific IgE was detected by Immunocap or by the skin prick or prick to prick test.

Food dependent exercise induced anaphylaxis (FDEIA) was diagnosed if anaphylaxis occurred during or immediately after exercise and when the patient had also consumed wheat based product just before or just after the exercise. Allergy to wheat was confirmed by the skin prick test with wheat, as well as in vitro testing for the detection of specific IgE to wheat or ω-5-gliadin (omega-5-gliadin). Wheat induced FDEIA was diagnosed if the patient had specific IgE to wheat or ω-5-gliadin, along with a relevant clinical history. All subjects who were considered to have FDEIA due to wheat could tolerate wheat based products during other times and developed reactions only if wheat was consumed before or just after exercise.

### Identification of anaphylaxis due to insects

Insect stings as a cause of anaphylaxis was confirmed when anaphylaxis occurred immediately following an insect sting and when the insect could be identified. Anaphylaxis to bee and wasp venom was confirmed by determining specific IgE to wasp and bee venom available in Western countries by Phadia ImmunoCap. Most episodes of anaphylaxis due to insects were following ant stings. However, testing for specific IgE for the insect could not be performed due to non-availability of commercial allergens. Levels of serum tryptase (Phadia ImmunoCap) were tested in all patients who developed anaphylaxis following insect stings 24 h or more after the episode of anaphylaxis, to exclude mastocytosis.

### Identification of vaccines or drugs as the possible cause of allergen

A drug or vaccine was identified as the cause of anaphylaxis when the patient developed anaphylaxis immediately after receiving the vaccine or after administration of the drug. All patients with vaccine induced anaphylaxis developed reactions within minutes after the immunization. Skin prick tests or challenge tests were not performed for possible anaphylaxis to the penicillin group of drugs, as this is discouraged in the recently updated WAO anaphylaxis guidelines [2].

In instances where the possible trigger of anaphylaxis could not be identified, such patients were considered to have idiopathic anaphylaxis.

### Severity of anaphylaxis

Severe anaphylaxis was evaluated according to the criteria defined by Brown [13]. Accordingly, those who developed hypotension or collapse, neurological compromise such as loss of consciousness or confusion, and hypoxia such as cyanosis or oxygen saturation of < 92% and incontinence were classified has having had severe anaphylaxis (grade 3).

## Results

**Fig. 1:**
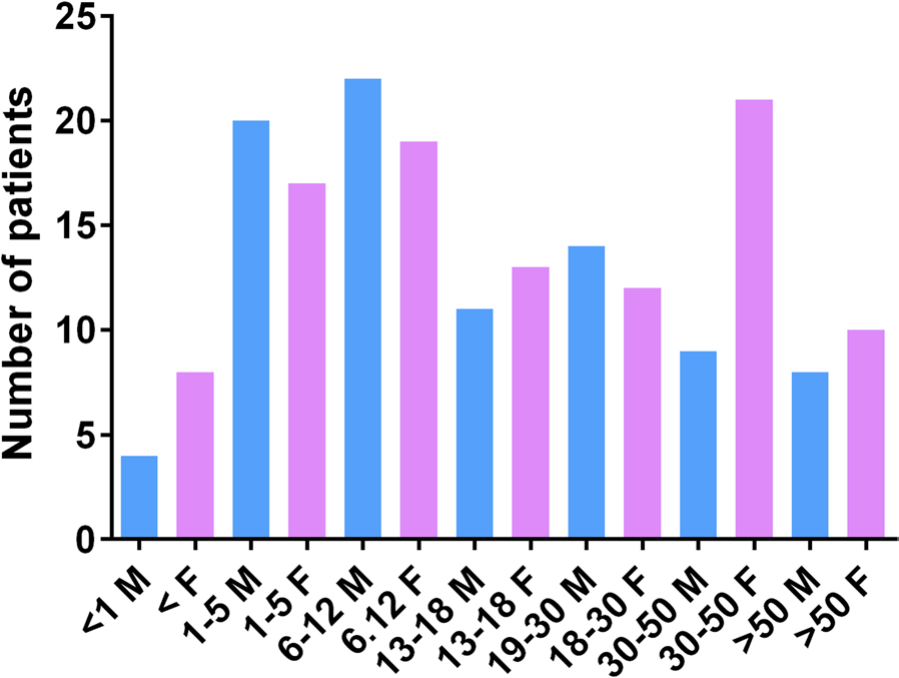
Age and gender distribution of 188 patients who presented with anaphylaxis. Males are represented by grey bars (M) and females are represented by black bars (F)

**Fig. 2:**
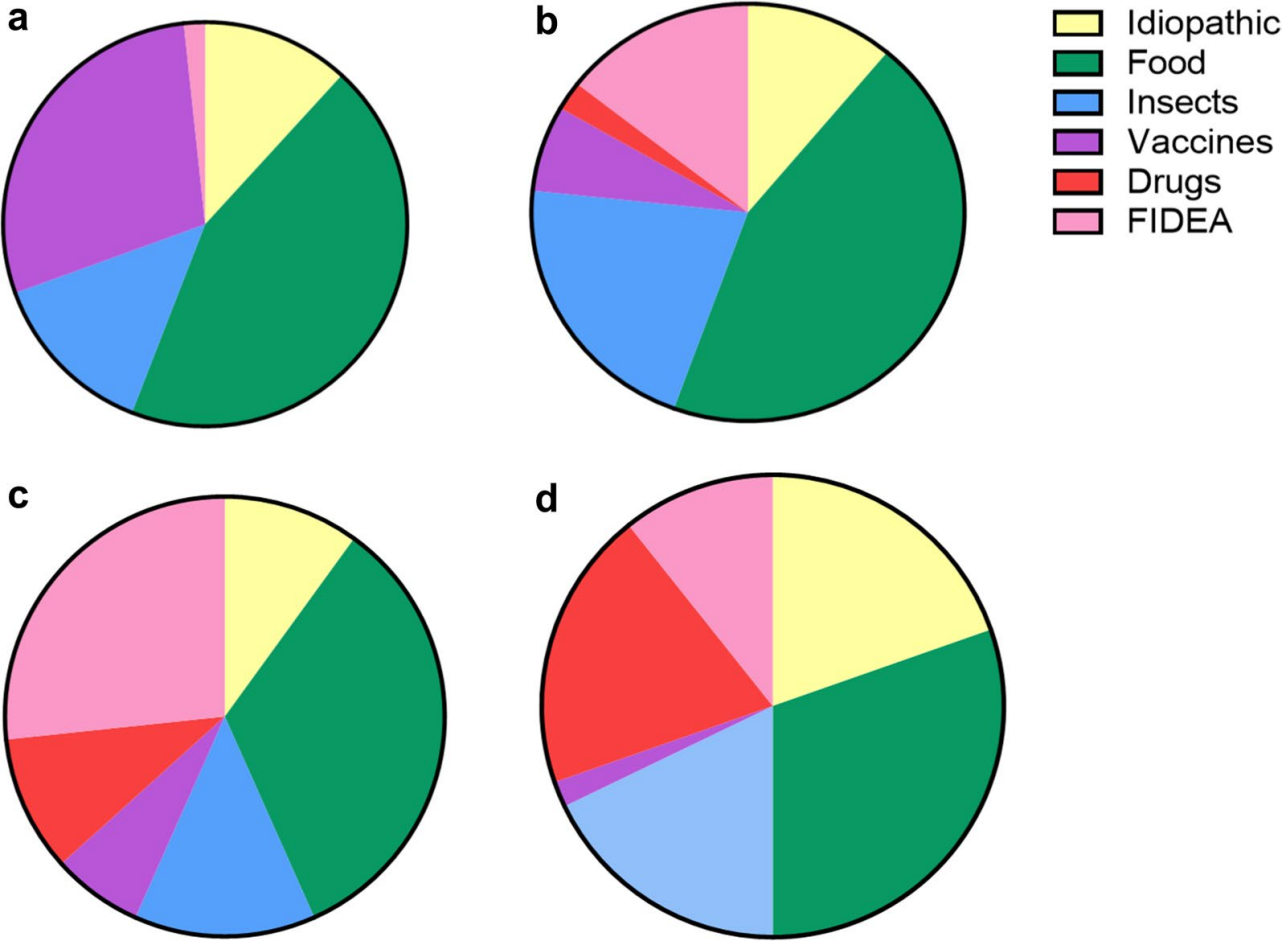
The aetiology of anaphylaxis in different age groups (n = 238). **a** In children < 5 years of age (n = 59), **b** in children between 5 and 18 years of age (n = 90), **c** in individuals between 19 and 30 years of age (n = 30) and **d** in individuals > 30 years of age (n = 56)

Two hundred and thirty-eight episodes of anaphylaxis were reviewed in 188 patients who were referred to our clinic (some patients had anaphylaxis to more than one allergen). The age and gender distribution of the cohort is shown in Fig. 1. Eighty-eight (46.8%) patients were males and 100 (53.2%) were females. Of the total cohort, 114 (60.6%) of those who developed anaphylaxis were children under the age of 18 years. Although equal proportion of males and females presented with anaphylaxis in childhood, anaphylaxis was more frequent among females (31/48, 64.5%) who were > 30 years of age. The causes of anaphylaxis identified in this cohort of patients are shown in Table 1. Anaphylaxis to food either following direct exposure 90/238 (37.5%) or after exercise in the form of FDEIA 29/238 (12.2%) was the predominant cause of anaphylaxis. Surprisingly vaccines accounted for 28 (11.8%) of instances of anaphylaxis, especially among children. The predominant cause of anaphylaxis changed with age, with food allergy being the most frequent trigger of anaphylaxis in childhood, while drug allergy and idiopathic anaphylaxis being more frequent after 30 years of age (Fig. 2).

**Table 1:** Causes of anaphylaxis

Cause of anaphylaxis	Number (%)
Idiopathic	31 (13.1)
Drugs	16 (6.7)
NSAIDS	4
Antibiotics	8
Drugs used in anesthesia	3
Eugenol	1
Vaccines	28 (11.8)
MMR	14
Japanese encephalitis	3
DT	2
DPT	1
Pentavalent	1
Hepatitis A and B vaccine	1
PCEC	3
FDEIA	29 (12.2)
Food	90 (37.5)
Cows’ milk	23
Beef	28
Pork	9
Mutton	6
Gelatin	2
Eggs	2
Shellfish	4
Cuttle fish	1
Fish	2
Wheat	3
Sesame	2
Miscellaneous	8
Insects	42 (17.7)
Formicidae (Ant species)	27
Apidae	3
Vespidae	8
Other	4
Other (hair dye and cold urticaria)	2 (0.0)

### Anaphylaxis due to food

Allergy to cow’s milk and red meat (beef/pork/mutton/lamb) accounted for 66/238 (27.7%) of instances of anaphylaxis and 66/90 (73.33%) of anaphylaxis due to food. All anaphylactic reactions to beef, pork, lamb and mutton in our cohort occurred within 1 h of exposure (in most patients within minutes). Delayed allergic reactions to red meat due to presence of IgE antibodies to the disaccharide galactose-α-1,3-galactose (α-Gal) [14] was not seen. Specific IgE to cow’s milk, beef, pork and mutton were detected by in vitro testing (Immunocap) or by skin prick testing in all patients who developed anaphylaxis following exposure to these foods. Two patients with allergy to beef also had an allergy to bovine gelatin. While all those with cow’s milk (CM) allergy were less than 12 years old, those with beef allergy belonged to all age groups. Ten of 44 (22.7%) episodes of anaphylaxis to red meats resulted in loss of consciousness (grade 3 anaphylaxis).

Although FDEIA is also a type of food allergy, it was classified as a separate entity as the patients could tolerate wheat based products when consumed in the absence of exercise. FDEIA was not detected in any patients below the age of 5 years and of the 29 patients, 21 (72.4%) were between the ages of 5–30 years. Seventeen of 29 patients (58.6%) developed severe anaphylaxis resulting in loss of consciousness. Two other patients in our cohort developed anaphylaxis for wheat without exercise and were not categorized under FDEIA. Another patient had exercise induced anaphylaxis, which was not related to the ingestion of food.

Only two patients developed anaphylaxis to cashew and peanut. Rare food items that triggered anaphylaxis in this cohort were mustard, mandarin, *Moringa oleifera* seeds, *Boerhavia diffusa* and *Manihot esculenta*.

### Anaphylaxis to vaccines

A large proportion of patients (11.8%) developed anaphylaxis following immunization. The majority of cases of vaccine associated anaphylaxis were due to the measles, mumps and rubella (MMR) vaccine, which contains bovine serum albumin. Of the 14 children who developed anaphylaxis to the MMR, 10 (71.4%) had allergy to CM and 5 (35.7%) were allergic to beef. One patient who did not have clinical CM allergy and had never consumed beef, had IgE to CM. Only one child, who developed anaphylaxis to the MMR did not have any allergy to CM or beef or specific IgE to either of these foods when tested by Immunocap. Three children had allergy to CM and beef, while another had allergy to only beef, and not to CM. Two of these children had anaphylaxis to the Japanese encephalitis (JE) and one to diphtheria tetanus (DT) vaccines as well.

Of the three children who developed anaphylaxis to the JE vaccine, two also had allergy to beef, and the other had allergy to CM. All three had IgE to beef, although only two had developed anaphylaxis to beef (the other had not consumed beef). Two of these children had also developed anaphylaxis to the MMR. The two individuals who developed anaphylaxis to the primary chick embryo cell (PCEC) vaccine against rabies were allergic to beef, and also had beef specific IgE antibodies. None of the individuals were allergic to eggs. As these children had developed anaphylaxis to vaccines, we did not carry out skin prick tests directly with the vaccine, as we thought the risks of this procedure outweighs the benefits to the patient.

### Anaphylaxis due to insects

Forty-two (17.6%) episodes of anaphylaxis occurred following insect stings or bites. Twenty-six of 42 (61.9%) insect stings occurred in males and males were more likely to develop anaphylaxis to insects compared to females, especially < 18 years of age (45.2% vs 14.2%). The majority of insects were of the order Hymenoptera, with ant species (family Formicidae) accounting for 27 cases, and *Apis cerana* (family Apidae), *Vespa affinis* and *Ropalidiya* species (family Vespidae) accounting for 11 patients. One infant developed anaphylaxis to a caterpillar, while another patient developed anaphylaxis on three occasions to a biting insect (Order Hemiptera), *Triatoma rubrofasciata* (family Reduviidae, kissing bug). The insect could not be identified in two patients.

**Fig. 3:**
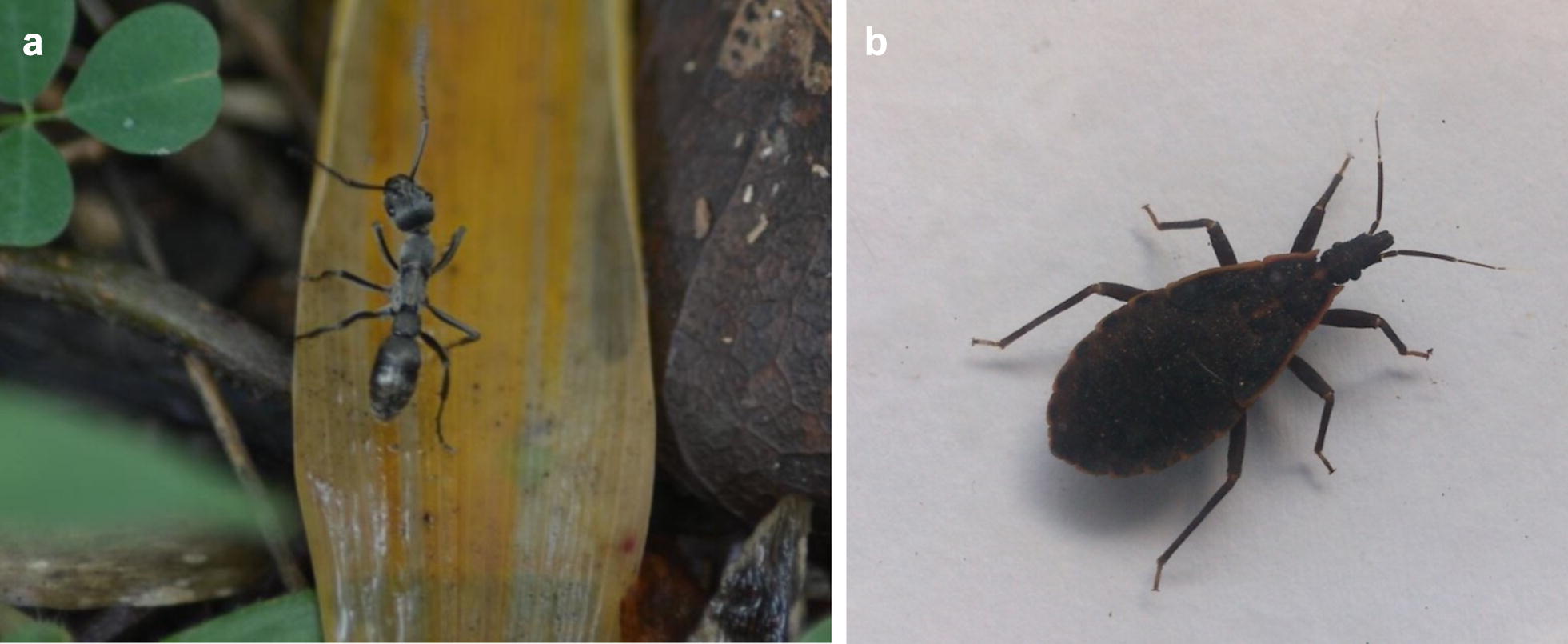
Insect species causing anaphylaxis. **a** *Diacamma rugosum*, **b** *Triatoma rubrofasciata*

The majority of anaphylaxis episodes following ant stings were due to *Odontomachus simillimus* followed by *Tetraponera rufonigra, Diacamma rugosum* (Fig. 3a) and in one instance due to *Solenopsis geminata*. Fourteen of the 27 episodes (51.8%) occurred in children aged 12 years or younger. Seven of the episodes lead to loss of consciousness, of which five were in children less than 12 years of age. Although anaphylaxis due to these ant species have been previously reported [15–17], there are no commercial tests to confirm allergy to these insects. Therefore, anaphylaxis to these insects was confirmed by identification of the insect that caused immediate reactions following the sting.

Of the 11 episodes of anaphylaxis due to bees and wasps, only 4 (36.3%) were in patients 12 years or younger. In one patient, anaphylaxis was triggered following a bite of the Triatominae species of insects (*T. rubrofasciata*, kissing bug) (Fig. 3b). Again, specific IgE could not be detected due to non-availability of commercial tests. Baseline serum tryptase levels were normal in all patients.

### Anaphylaxis due to drugs

Drugs (other than vaccines) accounted for 16 (6.7%) instances of anaphylaxis in this cohort of patients. Anaphylaxis due to antibiotics was the predominant cause followed by NSAIDs. Of the antibiotics that triggered anaphylaxis, beta lactam antibiotics were the predominant group (n = 6). Thirteen (81.2%) episodes of drug induced anaphylaxis occurred in those > 30 years of age. Anaphylaxis due to drugs were more frequent among females > 30 years of age (56.2%) compared to males of the same age (12.5%).

### Clinical features

Cutaneous symptoms such as generalized urticaria, angioedema and pruritus were seen in 209 (87.8%) episodes of anaphylaxis (Table 2). However, cutaneous symptoms were absent in 11 episodes of which seven were due to vaccines or anesthetic agents, and occurred within 5 min of exposure to the allergen. Symptoms and signs pertaining to the respiratory, cardiovascular and gastrointestinal symptoms were seen in 75.6%, 75.6% and 51.2% respectively. Severe anaphylaxis as defined as grade 3 anaphylaxis was seen in 81/238 (34%) episodes of anaphylaxis (Table 2). Hypotension was seen in 81 (34%), loss of consciousness (LOC) in 63 (26.5%) and hypoxia/cyanosis in 16 (6.7%) episodes. Occurrence of LOC during anaphylaxis appeared to increase in frequency with age (Table 3). For instance, LOC was seen in 29.8% of the episodes in those aged < 5 years, 47.8% aged between 13 and 18 years and 58.7% in those aged > 50 years of age. Interestingly, bradycardia was also seen in four episodes.

**Table 2:** Symptoms and signs of anaphylaxis

Clinical symptoms	N (%)
Cutaneous symptoms	227 (95.4%)
Urticaria	146
Angioedema	49
Flushing	05
Pruritus	77
No cutaneous symptoms	11 (5.2%)
Respiratory symptoms	180 (75.6%)
Shortness of breath	99
Rhonchi	14
Stridor	03
Rhinitis	08
Dysphonia	03
Hypoxaemia/cyanosis	16
Cough	31
Red eyes	05
No symptoms	43
Cardiovascular	177 (75.6%)
Hypotension	81
Tachycardia	18
Bradycardia	07
No pulse	10
Unrecordable blood pressure	15
Loss of consciousness	63
Incontinence	04
ECG changes	02 (ST elevation, T inversion)
Cardiac arrest	03
Chest pain	03
Cold peripheries	07
Blurred vision	05
No symptoms	38
Gastrointestinal	122 (51.2%)
Vomiting	46
Nausea	01
Diarrhoea	10
Dysphagia	02
Pain	27
No symptoms	90

**Table 3:** Symptoms of severe anaphylaxis in different age groups

Clinical symptoms	< 5 years N = 47 (%)	5–12 years N = 39 (%)	13–18 years N = 23 (%)	19–30 years N = 22 (%)	> 30 years N = 46 (%)
Hypotension	14 (29.8)	17 (43.6)	11 (47.8)	12 (54.5)	27 (58.7)
Loss of consciousness	11 (23.4)	12 (30.8)	14 (60.8)	7 (31.8)	21 (45.6)
Unrecordable blood pressure	1 (2.1)	6 (15.4)	5 (21.7)	1 (4.5)	2 (9.1)
Bradycardia	2 (4.2)	0	3 (13.0)	0	2 (9.1)
Cardiac arrest	1 (2.1)	1 (2.6)	0	0	1 (4.5)
Absence of pulse	4 (8.5)	2 (5.1)	2 (8.7)	0	2 (9.1)
ECG changes	0	0	1 (4.3)	1 (4.5)	0
Severe hypoxia/cyanosis	6 (12.8)	4 (10.2)	2 (8.7)	3 (13.6)	1 (2.2)

### Treatment of anaphylaxis

Data regarding treatment of the episode of anaphylaxis was available for only 104/238 episodes that were assessed in our clinic, as the treatment given was not recorded in the other instances. Adrenaline was administered intramuscularly in 75 (72.1%) of episodes, administered as an intravenous bolus on two occasions, intravenous infusion in two occasions, in the form of a nebulization in two occasions and subcutaneously on one occasions. Adrenaline was not administered in 26 (25%) of instances.

## Discussion

In this study we have reviewed 238 episodes of anaphylaxis in 188 patients who were referred to an immunology clinic in Colombo, Sri Lanka. This study highlights the differences in the aetiology of anaphylaxis in different countries. The commonest cause of anaphylaxis in this cohort was food allergy, as reported in many other studies [3, 8, 18]. Food induced anaphylaxis accounts for 50–80% of instances of anaphylaxis in children and approximately 200 deaths per year are thought to occur in the US due to anaphylaxis to food [19]. However, the types of food implicated as the predominant cause of anaphylaxis varies with each geographical region possibly due to differences in weaning practices and food preparation. For instance, in Europe the commonest foods that triggered anaphylaxis were cow’s milk, tree nuts, peanuts and egg [18], whereas in Tennessee, United States the commonest foods were shell fish, peanuts, tree nuts and food additives or spices. In our cohort, the commonest food that triggered anaphylaxis in very young children was cow’s milk whereas it was beef in older children and adults.

Anaphylaxis to beef accounted for 36.5% of the instances of anaphylaxis to food. Although anaphylaxis to beef has been reported in other studies [8], it is rare [20]. Anaphylaxis to beef due to sensitization to the disaccharide galactose-α-1,3-galactose (α-Gal), is being increasingly reported [14, 21]. In instances where anaphylaxis occurs due to α-Gal, the onset of anaphylaxis is often delayed for 5–6 h and the specific IgE for beef cannot be detected by skin prick tests or by in vitro tests (ImmunoCap). In our patient cohort all patients developed immediate anaphylaxis to beef and specific IgE to beef was detected in all of them. The high incidence of beef allergy appears to also have implications in vaccine related anaphylaxis. For instance, 10/14 individuals who developed anaphylaxis to the MMR vaccine and all those who developed anaphylaxis to the Japanese encephalitis vaccine had specific IgE to beef (only two had anaphylaxis and three had an allergic reaction). The two patients who developed anaphylaxis to the PCEC and to the Pentavalent vaccine also had specific IgE to beef. Although specific IgE to beef was detected in the majority of individuals who developed anaphylaxis to vaccines, many of them had not consumed beef and only a few had either anaphylaxis or symptoms of allergy when consuming beef. Since very small amounts of bovine serum albumin is present in many vaccines including the MMR [22], such ingredients in vaccines are likely to cause anaphylaxis in those who are sensitized to beef [10]. The proportion of individuals who developed anaphylaxis following vaccines was quite high in our study (11.8%). Since all vaccine associated adverse reactions including anaphylaxis are reported and thoroughly investigated by the Sri Lankan authorities, we believe cases of vaccine associated anaphylaxis may be over represented in our patient cohort due to referral of all such vaccine induced anaphylaxis patients to our unit.

Quite a large proportion of individuals (12.2%) in our cohort had FDEIA to wheat, which was confirmed by either skin prick tests or in vitro tests. Importantly, a large proportion of patients who developed FDEIA had grade 3 anaphylaxis (58.6%) and had repeated episodes, as the cause could not be identified by the medical personal or the patients. The high incidence of FDEIA in our study cohort could be due to referral of such patients by other clinicians as the cause of anaphylaxis could not be identified. Neither the referring clinicians nor the patients had thought of wheat as a possible cause of their anaphylaxis, although most patients had noted that they developed anaphylaxis following exercise.

Anaphylaxis due to insect stings accounted for 17.7% of cases in our cohort. The predominant cause of insect venom anaphylaxis was due to allergy to two main ant species, *O. simillimus* and *T. rufonigra.* Anaphylaxis due to the ant *O. simillimus* has been reported in Sri Lanka [17], while anaphylaxis due to *T. rufonigra* has been reported in other South East Asian countries and in Sri Lanka [15, 17]. To the best of our knowledge, allergy to stings of *D. rugosum* has not been reported. The commonest species causing Hymenoptera venom anaphylaxis belonged to the genus *Ropalidia*. In a different study carried out in the Southern region of Sri Lanka where there are numerous tea estates, the commonest insect causing Hymentoptera allergy was *Apis dorsata* and by *Vespa tropica* followed by *Ropalidia marginata* [6]. Anaphylaxis due to *T. rubrofasciata* has been reported in the Americas, but is rare in Asia, with two case reports from Singapore [23] and Hawaii [24]. Our patient is the first reported in the Indian subcontinent. *T. rubrofasciata* infestation is a growing menace in Asia [25], and an increase in allergic reactions is likely.

Systemic reactions to insect stings in children are mild and generally cutaneous compared to adults [22, 26]. Hypotension and loss of consciousness are rare in children [27]. The majority of our patients, including children who developed anaphylaxis following ant stings developed LOC. This was in contrast to stings by bees and wasps in our study. The epidemiology and clinical features due to ant stings appear to be different following wasp and bee stings and therefore needs to be further studied.

As reported in many other studies, drug induced anaphylaxis was more frequent among older individuals and were predominantly due to antibiotics followed by NSAIDs [5, 8]. Due to the possibility of triggering anaphylaxis and since skin prick tests and challenge tests are now not recommended for penicillin allergy [2], we only identified that the drugs as a cause of anaphylaxis based on the patients’ history. As reported in previous studies, the cause of anaphylaxis could not be identified in a large proportion (15.7%) of individuals [8] and these individuals were classified as having idiopathic anaphylaxis.

## Conclusions

We have identified the causes of anaphylaxis in 188 patients who had 238 episodes of anaphylaxis. The commonest cause of anaphylaxis was food followed by vaccines and insect venom. The predominant food items and insects responsible for anaphylaxis were markedly different to those of Western countries.
